# Architecture and sequence stratigraphy of the Upper Coralline Limestone formation, Malta—Implications for Eastern Mediterranean restriction prior to the Messinian Salinity Crisis

**DOI:** 10.1002/dep2.138

**Published:** 2021-03-02

**Authors:** Or M. Bialik, Raymond Zammit, Aaron Micallef

**Affiliations:** ^1^ Marine Geology & Seafloor Surveying Department of Geosciences University of Malta Msida Malta; ^2^ School of Earth and Environmental Sciences Cardiff University Cardiff UK; ^3^ GEOMAR Helmholtz Centre for Ocean Research Kiel Kiel Germany

**Keywords:** carbonate platform, Central Mediterranean, Late Miocene C‐type carbonate factory, Malta Plateau

## Abstract

The Eastern and Western Mediterranean are separated by an elevated plateau that regulates water exchange between these two basins. The Maltese archipelago, situated atop this topographic high, offers a unique window into the evolution of this plateau in the lead up to the Messinian Salinity Crisis. The Upper Coralline Limestone Formation was deposited between the late Tortonian and the early Messinian and was probably terminated by palaeoceanographic events related to the Messinian Salinity Crisis. It represents the youngest Miocene sedimentary deposits outcropping in the Maltese archipelago. This shallow‐water carbonate unit can be used to trace palaeoenvironmental changes atop the sill between the Eastern and Western Mediterranean and to explain the possible water flow restrictions to the Eastern Mediterranean that could have preceded the Messinian Salinity Crisis. Here field surveys, and analysis of the depositional environment within the Upper Coralline Limestone in Malta, are combined with recently acquired multichannel seismic reflection profiles between Malta and Gozo, to reconstruct the depositional sequence in the Malta Plateau during the late Miocene. The Upper Coralline Limestone consists of multiple coralline and larger benthic foraminifera dominated facies, extending from subtidal to intertidal environments. These accumulated in two depositional cycles observed in both outcrop and seismic reflection data. Each cycle exhibits an early aggradation–progradation phase followed by a progradation phase and a final aggradation phase. These manifest themselves in the outcrops as shallowing and deepening upwards phases. These were deposited above a deep water unit and are indicative of a preceding uplift phase followed by filling of the accommodation space through the deposition of the Upper Coralline Limestone Formation in shallow marine depths. The presence of this highly elevated sill during the late Miocene could have restricted circulation to the eastern basin.

## Graphical abstract

The Upper Coralline Limestone formation in Malta offers a unique look at the gateway between the West and East Mediterranean in the period before the Messinian Salinity Crisis (MSC). Here we revisit this unit using both field bases sedimentological analysis and seismic imaging to document the evolution of this sequence. The findings illustrate the ongoing uplift of the barrier between the basins before the MSC.

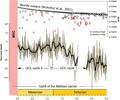

## 1 INTRODUCTION

The Messinian salinity crisis (MSC, 5.97–5.33 Ma) was the result of extreme restriction of the Mediterranean Sea from the Atlantic Ocean (Hsü et al., 1973; Meilijson et al., 2019; Roveri et al., 2014a). Yet the impact and effect of the MSC were not uniformly distributed. The Eastern Mediterranean hosts around 70% of total MSC salt deposits, in significantly thicker deposits than the Western Mediterranean (Haq et al., 2020), indicating a marked difference in hydrology during the MSC. While it is accepted that evaporite deposition was synchronous in both the Western and Eastern Mediterranean (Krijgsman et al., 2002; Meilijson et al., 2018, 2019), an ongoing debate persists on whether the Eastern and Western Mediterranean were restricted to the same degree and at the same times (Blanc, 2000, 2006; Meijer, 2006; Ryan, 2008). In the aftermath of the MSC (the Zanclean flood), the western basin was the first to be reflooded. Subsequent flooding of the Eastern Basin occurred via a massive waterfall over the Maltese Escarpment due to overflow from the Western Basin (Garcia‐Castellanos et al., 2020; Spatola et al., 2020).

The ^87^Sr/^86^Sr isotope ratio of carbonate deposits in the Eastern Mediterranean exhibit a departure from oceanic values, indicating modification and possible restriction from the world's oceans already during the late Tortonian (Schildgen et al., 2014). Reef accumulation patterns in the Eastern Mediterranean diverge from that of the Western Mediterranean, both in number and composition (Buchbinder, 1996), notably in respect to coral assemblages and presence. Reduction of availability of cold oceanic waters was suggested as playing a role in this (Coletti et al., 2019; Esteban, 1979). Microfossil assemblages exhibit indications of periodically enhanced salinity and oxygen limitation (Kontakiotis et al., 2019; Kouwenhoven et al., 2006; Moissette et al., 2018). All of this evidence points to some enhanced restriction of the eastern basin as early as *ca* 7.5 Ma.

A key region controlling the connection between the Eastern and Western Mediterranean is the sill between Sicily in the north and Tunisia in the south. This sill is part of the North African continental margin (Jongsma et al., 1985). The region is tectonically complex where rifting, transpression and the African/European collision resulted in a highly dynamic configuration throughout the Miocene (Ben‐Avraham et al., 1987; Dart et al., 1993; Gardiner et al., 1995). This activity allowed for significant vertical movements, well expressed in the geological record of the Maltese Islands by significant shifts in depositional depth (Pedley, 1978). In this study, the focus is placed on the sedimentary evolution of this area and, mainly, on the depositional sequence of the late Tortonian and Messinian ages, prior to the MSC. The aim here is to study and delineate the evolution of the sequence during this time. By integrating outcrop and seismic reflection data, it is hoped to explore possible indications of vertical fill that could have resulted in the restriction of water exchange between the Eastern and Western Mediterranean.

## 2 GEOLOGICAL SETTING

The Maltese archipelago is a relic of an ancient carbonate platform located in the north‐eastern part of the Pelagian Block and initially formed during the Palaeogene (Gatt & Gluyas, 2012; Micallef et al., 2016). The greater structure dates back to the early stages of the Tethys Ocean, and since then has been relatively higher than its surroundings (Jongsma et al., 1985). During the late Miocene, this area experienced further uplift driven by SE‐NW directed horizontal shortening as plate convergence between Africa and Europe changed the regional tectonic stress field (Adam et al., 2000; Gutscher et al., 2016; Reuther et al., 1993). These large magnitude vertical shifts of the Malta Plateau are well illustrated in the late Miocene by the transition from the hemipelagic Serravallian Blue Clay Formation to the shallow‐water carbonates of the Tortonian–Messinian Upper Coralline Limestone (UCL) Formation, which are separated by a glauconitic unit referred to as the Greensand Formation (GS, Baldassini & Di Stefano, 2017; Catanzariti & Gatt, 2014).

The UCL is a calcareous shallow marine succession and the youngest Miocene rock unit exposed in the Maltese Archipelago (Pedley, 1978). The age of the base of the UCL had been recently assigned to the upper part of Nannofossil zone MNN11a (CNM16, *ca* 8 Ma; Baldassini & Di Stefano, 2017), while the age of its uppermost part is unknown and inferred to be either shortly after the MSC (Pedley, 2011) or within the early part of the MSC (Cornée et al., 2004). Early interpretation, based on sedimentary components and modern coralline algae, described the deposition of the UCL as a shallowing upward sequence (Pedley, 1978; Table 1). The UCL does not exhibit any of the extreme stress features reported in the Calcare di Base (Borrelli et al., 2020), which marks the onset of the MSC in Sicily. Thus, it probably represents more moderate temperature/salinity conditions. The geometry of the UCL was inferred to be a belt of coralline bioherms (Bosence & Pedley, 1982; Pedley, 1976) aligned SE‐NW along the southern margin of the island, and developing atop a palaeorelief. The individual beds described are representative of different lateral and temporal positions with respect to a carbonate platform (Cornée et al., 2004). Nevertheless, the described elements delineate the depositional depth of the UCL to <100 m in the deeper parts, and intertidal depths in the shallowest parts.

**TABLE 1:** Original subdivision of the UCL (Bosence & Pedley, 1982; Pedley, 1976, 1978) to members and beds, their assigned depositional settings and water depth

Member	Bed	Sub bed	Thickness (m)	Water depth	Environment
Gebel Imbarkk	Qammieħ		25	Intertidal to very shallow subtidal (0–*ca* 5 m)	Restricted intertidal to shallow marine (coastal lagoon?)
	Tat‐Tomna		2.3	Intertidal (0–1 m)	Open intertidal to shallow marine
Tal‐Pitkal	Depiru	Għadira	>23	Intertidal to very shallow subtidal (0‐*ca* 5 m)	Open intertidal to shallow marine
		Għar Lapsi	25	Intertidal to very shallow subtidal (0‐*ca* 5m)	Intertidal to shallow marine, pos. restricted
		Sensu*stricto*	3	<25 m	Open marine shallow waters *1 m marl layers are present
	Rabat Plateau		?	12–15 m	Open marine shallow waters
Mtarfa	Rdum il‐Ħmar		12	<100 m	Mid shelf open marine
	Gebel Mtarfa		7		Mid shelf open marine
		Coralline Algal bioherm	16	>50 m	Mid shelf open marine
Għajn Melel	Zebbug		16.5	<50 m	Mid shelf open marine
	Għajn Znuber		5.1	Sublittoral (<200 m)	Mid to outer shelf open marine, Pos. at interaction with OMZ

## 3 METHODS

### 3.1 Field data

**FIGURE 1:**
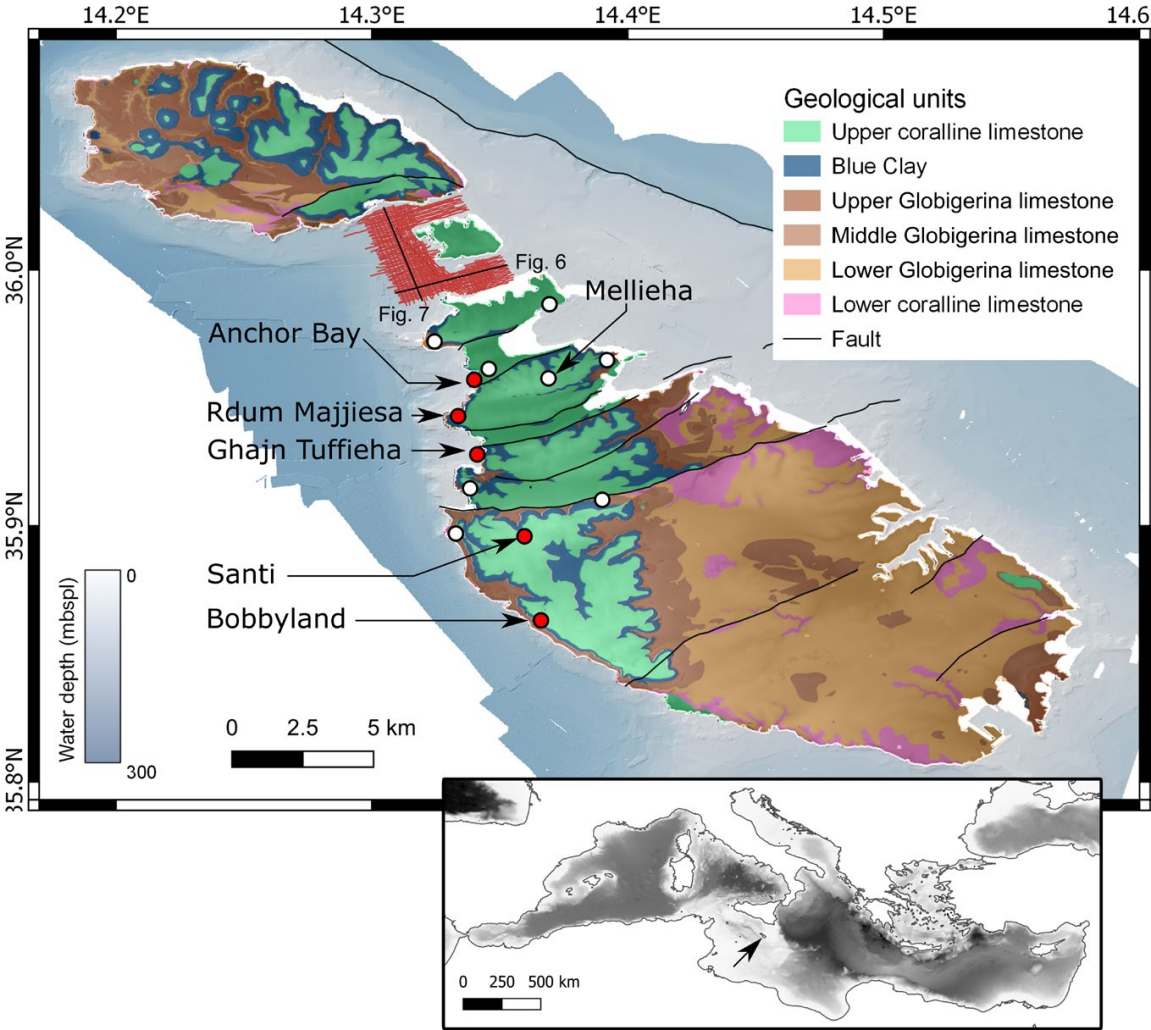
Geological map of the Maltese archipelago showing the location of the study sites and area covered by the seismic survey. Location of outcrops shown in Figure 2 and columnar sections shown in Figure 3 are noted in red with names. Arrow in the inset map shows the location of the Maltese archipelago in the modern Mediterranean

Fieldwork in 2017 included a systemic survey of UCL outcrops on the western side of the island of Malta. Five outcrops were chosen for study (from north to south, Figure 1): Anchor Bay (35.93°N/14.34°E; 13m), Rdum Majjiesa (35.94°N/14.33°E; 18m), Għajn Tuffieħa (35.93°N/14.34°E; 8.5m), Santi (35.82°N/14.36°E; 16m) and Bobbyland (35.85°N/14.37°E; 4m). Additionally, outcrops at St. Paul's Bay; Rdum il‐Qammieħ; Ras il‐Pellegrin; Mellieħa; the range between Blata tal‐Melħ and Rdum tal‐Vigarju; Rdum il‐Ħmar and the Bingemma syncline (white markers in Figure 1) were visited to examine the appearance and larger scale geometries. Facies description at the outcrop‐level of the unit was done using the modified Dunham (1962) classification by Embry and Klovan (1971). These outcrops were selected to represent a near‐complete picture of the UCL depositional sequence in Malta; they contain most of the known facies of the UCL in the island of Malta except for the ‘Qammieħ beds’ facies (Pedley, 1978), which does not outcrop in any of the surveyed sites. Some 96 samples were collected and analysed using an XRD Rigaku MiniFlex 600 benchtop X‐ray diffractometer (30 kV/10 mA from 3° to 70° at 0.05° increments by point detector) to test viability for geochemical analysis.

### 3.2 Geophysical data

Seismic interpretation was carried out on 240 km of multichannel seismic reflection profiles from the Gozo Channel project (Figure 1, more detailed image shown in Figures S1–S3) using a mini GI gun with a total volume of 1 L (60 cu. in.). A shot point distance of 15.625–18.750 m and a recording length of 2 s were used. Data were recorded using a 300 m long digital streamer with 96 channels, with a channel distance of 3.125 m. The fold coverage ranged between 8 and 9.6 traces per common depth point (CDP). The processing sequence included amplitude recovery, band‐pass filtering, CDP sorting, pre‐stack deconvolution, velocity analyses, normal move out correction and stacking.

Based on existing knowledge (Pedley, 1976), this investigation has been limited to the upper 200 ms of the seismic data. The sequence has been subdivided into Seismic Units (SUs) based on unconformities and seismic facies. These SUs were numbered from bottom to top.

## 4 RESULTS

### 4.1 Outcrops

#### 4.1.1 Anchor Bay

**FIGURE 2:**
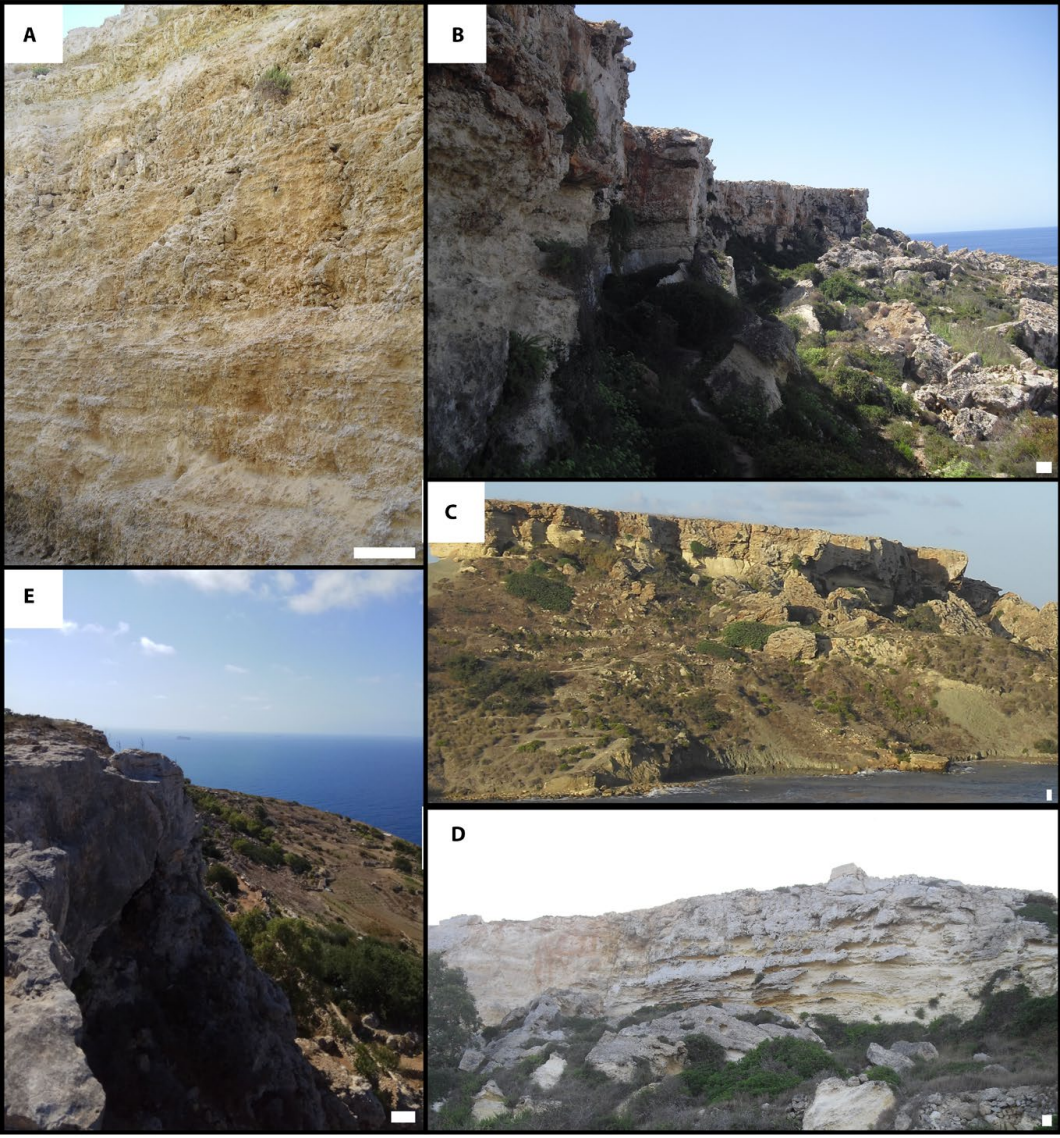
Overview photographs of the outcrops logged (clockwise): (A) Anchor Bay; (B) Rdum Majjiesa; (C) Santi; (D) Għajn Tuffieha; (E) Bobbyland. White scale bar is 1 m

**FIGURE 3:**
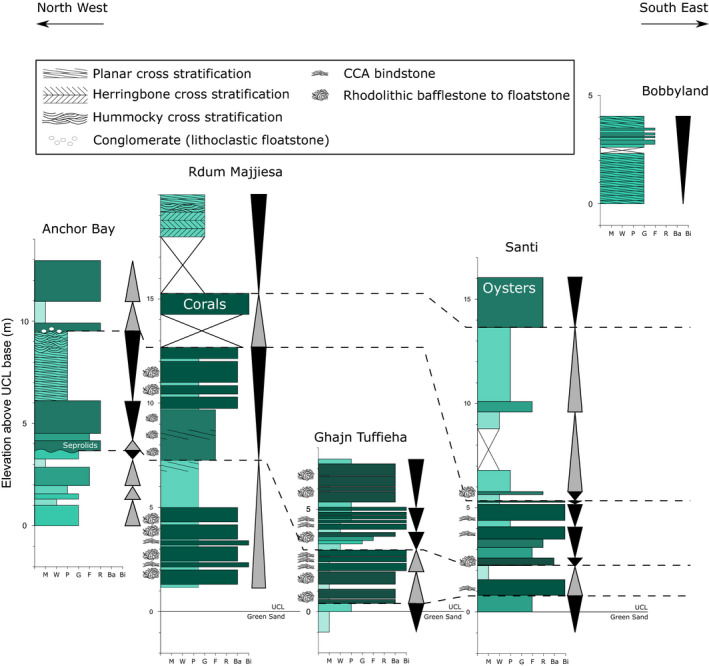
Columnar sections of the investigated outcrops showing the present facies and features in each locality. The horizontal axis for each column indicates the Embry and Klovan (1971) class of each part of the section. M—Mudstone; W—Wackestone; P—Packstone; G—Grainstone; F—Floatstone; R—Rudstone; Ba—Bafflestone; Bi—Bindstone

**FIGURE 4:**
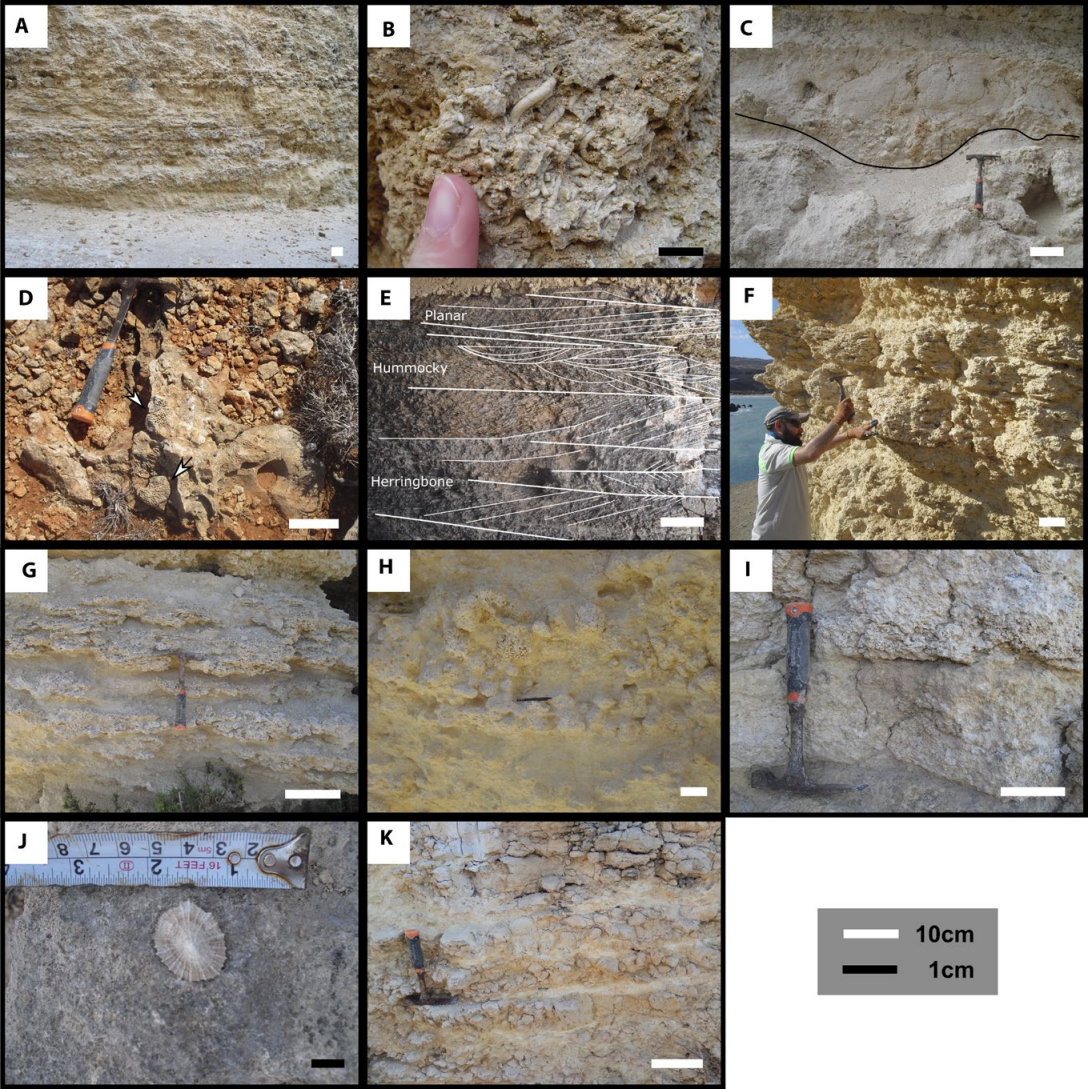
Facies occurrence in the UCL: (A) Alternating packstone and grainstone layers, Anchor Bay; (B) serpulid bafflestone, Anchor Bay; (C) channel fill conglomerate (Intraclastic floatstone, clasts comprised of grainstone), Anchor Bay; (D) coral beds, corals are noted by arrows, Rdum Majjiesa; (E) alternating cross‐stratification grainstone at the top of the Rdum Majjiesa section; (F) alternating bafflestone layers and mudstone to wackestone, Għajn Tuffieħa; (G) alternating bindstone and wackestone, Għajn Tuffieħa; (H) rhodolithic floatstone, Santi; (I) coralline packstone to floatstone exhibiting weak cross‐stratification, Santi; (J) Limpet on background of a packstone layer, Santi; (K) imbricated lithoclastic (clasts comprised of grainstone) floatstone, Bobbyland

The Anchor Bay outcrop (Figures 2A and 3) exposes *ca* 13 m of the UCL. The outcrop is in a channel excavated during road construction leading into a local embayment. The base of the outcrop is above the GS/UCL contact; the contact can be identified underwater in the embayment. The lower 3.5 m of the section consist of alternating layers of fine‐grained facies mudstone/wackestone/packstone and coarser‐grained grainstone/floatstone rich in mixed coralline algae and large benthic foraminifera (LBF) facies (Figure 4A). This initial section is truncated by an erosion channel identified in this outcrop. This channel is filled with coarse‐grained intraclasts, and serpulids (Figure 4B) have been identified at the base of the channel. Floatstone/rudstone facies are present up to 6 m, above which the facies change to planar cross‐stratified packstones capped by hummocky cross‐stratified layers terminated by the second erosion surface identified at Anchor Bay. The infill floatstone layer is capped by a mudstone layer (Figure 4C), and the section terminates with 2 m of coarse‐grained material. All samples analysed from this outcrop were >90% low‐Mg calcite.

#### 4.1.2 Rdum Majjiesa

The Rdum Majjiesa section (Figures 2B and 3) outcrops in the Majjistral National Park (Figure 1) and comprises around 20 m of exposed UCL. Just like in the Anchor Bay section, neither the GS Formation nor the base of the UCL has been identified in the field. The lower 5 m of the section is composed of layers of thin, centimetre‐scale mudstone/wackestone beds alternating with thicker crustose coralline algae (CCA) bindstone and rhodolithic bafflestone to floatstone facies. The rhodoliths are 5–10 cm in diameter, and are for the most part spherical and well‐rounded; smaller (0.5–2 cm) coralline nodules are present and bound in the CCA beds. Solitary corals were also encountered in these bed sets. The mid‐part of the section consists of *ca* 5 m of mixed coralline algae and LBF packstone to grainstone beds. Planar cross‐stratification is present in these beds, which are then followed by another sequence of mudstone/wackestone alternating with CCA bindstone and rhodolithic bafflestones to floatstone. This sequence is terminated by an unconformity. A coral (*Porites?*; Figure 4D) bed has been identified at *ca* 15 m from the base of the section and the section terminates with 2 m of herringbone to planar to hummocky cross‐stratified coralline algae and LBF packstone/grainstone beds (Figure 4E). All samples analysed from this outcrop were >90% low‐Mg calcite.

#### 4.1.3 Għajn Tuffieħa—Qarraba

The base of the outcrop at Għajn Tuffieha Bay and Qarraba Bay (Figure 2C) expresses a gradual contact from the underlying GS Formation consisting of *ca* 1 m of glauconitic mudstone facies, which transitions into a *ca* 40 cm packstone bed representing the base of the UCL. Following this bed, the outcrop is dominated by coarse‐grained beds of rhodolithic floatstone to bafflestone and CCA bindstone interspersed by thin mudstone/wackestone beds (Figure 4F,G). The rhodoliths are 5–10 cm in diameter and are for the most part, spherical and well‐rounded. As of *ca* 7.5 above the base of the section, all material was heavily karstified and recrystallised. All samples analysed from this outcrop were >90% low‐Mg calcite.

#### 4.1.4 Santi

The Santi section outcrops (Figure 2D) along the country road near the village of Santi, *ca* 16 m of the UCL are exposed in this location above a visible contact with the GS Formation The base of the section consists of a grainstone bed that transitions into a *ca* 4 m layer of CCA bindstone, rhodolithic bafflestone to floatstone (Figure 4H) and some layers of wackestone/packstone of varying thicknesses. Weak cross‐stratification is observed at times (Figure 4I). The rhodoliths are 5–10 cm in diameter and are for the most part, spherical and well‐rounded. The mid‐part of the section consists mostly of LBF and mixed coralline algae packstone to grainstone facies. This part of the sequence is capped by an oyster‐rich horizon that also contains limpets (Figure 4J). Given that this outcrop is farther inland than the other outcrops and that these components were found embedded, they are considered to be original. All samples analysed from this outcrop were >90% low‐Mg calcite.

#### 4.1.5 Bobbyland

The Bobbyland outcrop along Dingli Cliffs (Figure 2E) contains 4 m of carbonate facies from the top of the UCL. These units have some similarities to the upper beds observed at Rdum Majjiesa and consist mostly of LBF and coralline algae planar cross‐stratified beds (Figure 4K). All samples analysed from this outcrop were >90% low‐Mg calcite.

#### 4.1.6 General geometry

**FIGURE 5:**
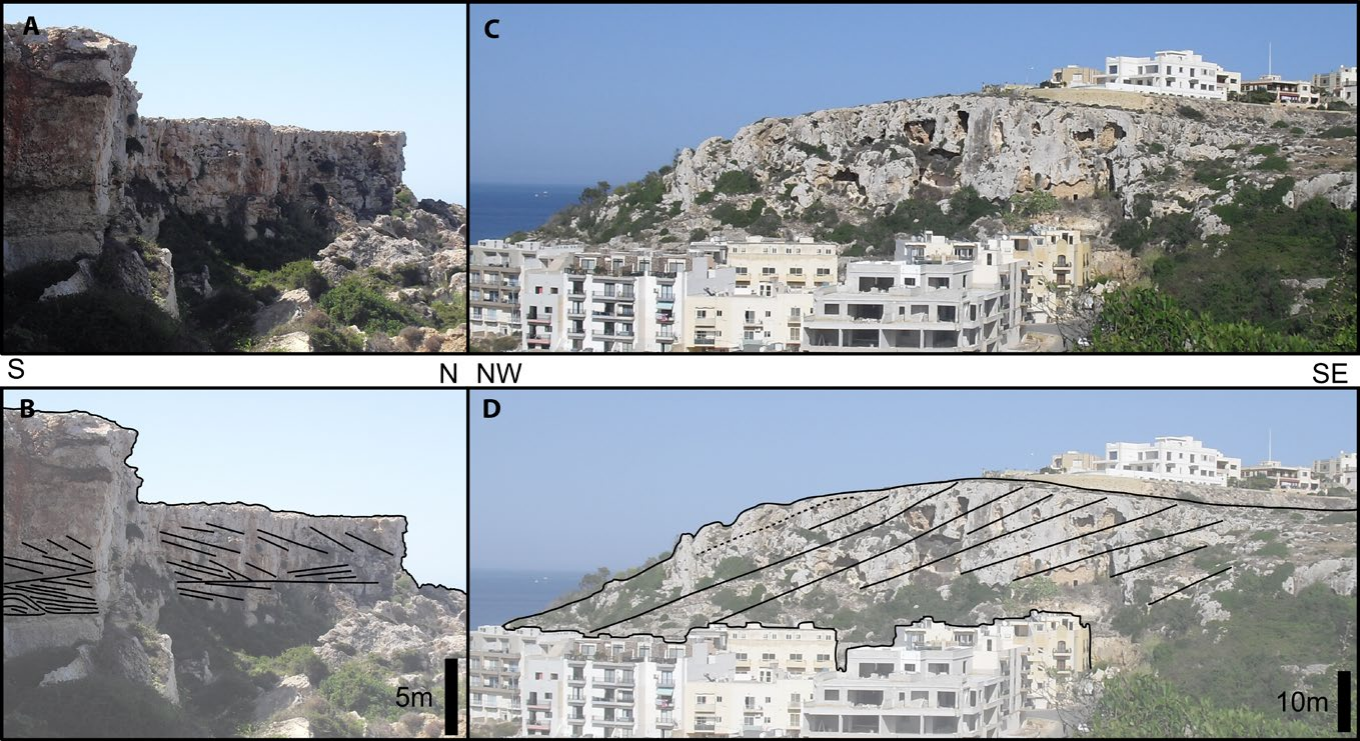
Large‐scale sedimentary features in the UCL. (A) Unretouched photograph and (B) bedding highlights show large inclined bedding at Rdum Majjiesa. (C) Unretouched photograph and (D) bedding highlights show large‐scale clinoforms at Mellieħa

The deposition geometry at the outcrop‐level is poorly expressed. Beds appear to be relatively continuous along a NW–SE trend but less so in the perpendicular direction. Where cross‐stratification was observed, both the herringbone and the planar stratification were oriented NE–SW. In none of the surveyed sites were lateral transitions of the coralline facies observed. Some outcrops exhibit well‐pronounced clinoforms, either as small bedding‐scale features dipping to the north to north‐east as observed at Rdum Majjiesa (Figure 5A,B), or large‐scale clinoforms dipping towards the north to north‐west as observed at Mellieħa (Figure 5C,D). At Rdum Majjiesa, these clinoforms were comprised of rhodolites, coralline nodules, coralline or LBF sand.

#### 4.1.7 Seismic reflection data

Within the designated study interval, five seismic facies were identified. SF1—High amplitude continuous reflections with downlap or onlap configuration; SF2—chaotic low amplitude reflections; SF3—low to medium amplitude inclined, clinoform‐like reflections, often expressing downlap; SF4—low to medium amplitude sub‐horizontal non‐continuous reflections, often exhibiting ponding and onlap geometries; SF5—medium amplitude horizontal to sub‐horizontal continuous reflections.

**FIGURE 6:**
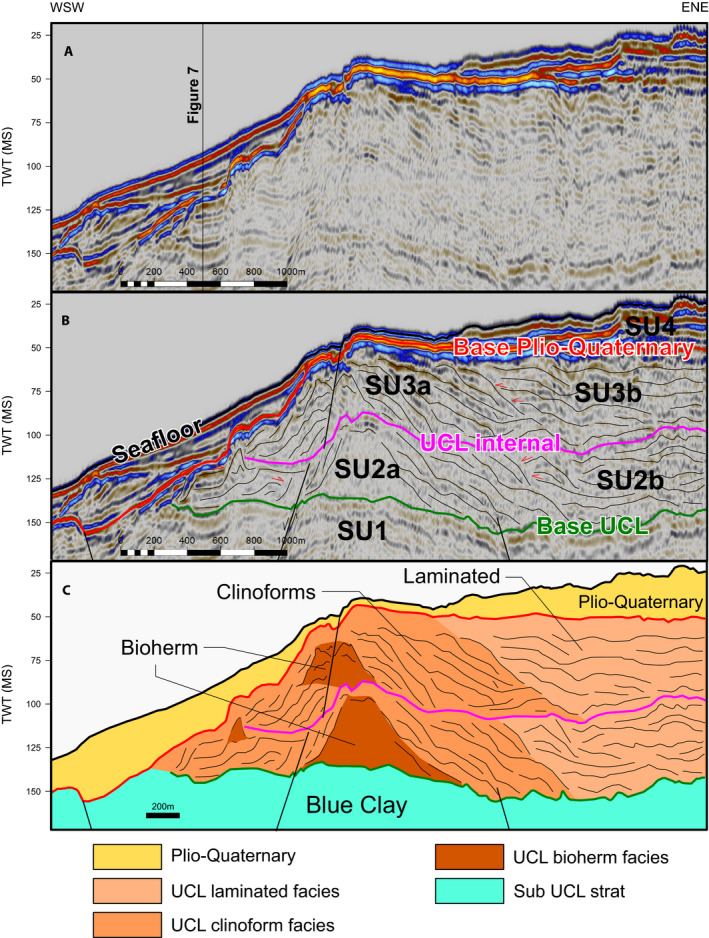
Uninterpreted (A) delineated (B) and interpreted (C) time‐migrated section. (A) Line MGT16_77 trending approximately WSW‐ENE. Seismic facies are shown in (C); SF1 in yellow, SF2 in dark orange, SF3 in light orange SF4 in pink and SF5 in blue

**FIGURE 7:**
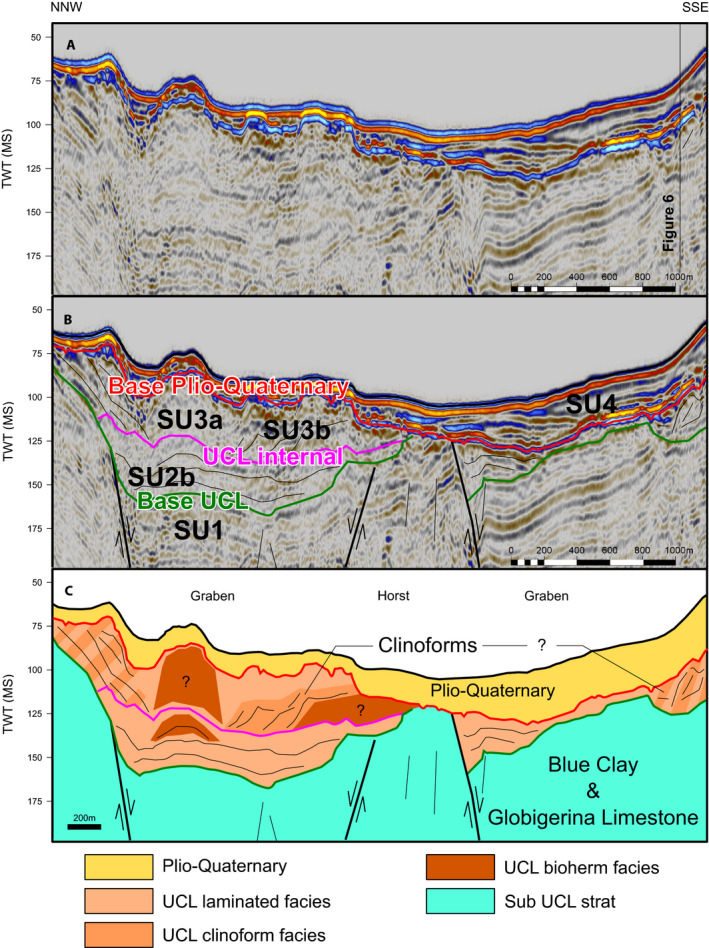
Uninterpreted (A) delineated (B) and interpreted (C) time‐migrated section. (A) Line MGT16_106 trending approximately NNW‐SSE. Seismic facies are shown in (C) SF1 in yellow, SF2 in dark orange, SF3 in light orange and SF4 in blue

The seismic facies relationships are best represented along a shore‐parallel transect between the islands (Figure 6). SF5 characterises the lower part of the investigated interval. It terminates to the south by a truncation and to the north by a pronounced strong reflection. The extent of this facies was delineated as SU1 (Figure 6B). Above this unit are lateral transitions between SF2, SF3 and SF4 (Figure 6C), capped by a pronounced reflection above which this pattern is repeated. These lateral elements were delineated as SU2 and SU3, with the SF2 and SF3 as the ‘a’ subunit and SF4 as the ‘b’ subunit. All units are truncated by a strong reflection above which SF1 is dominant; this was selected as the base of SU4, which extends above it until the sea floor. Unit thickness is not consistent, increasing between the islands and deceasing towards the shore, and conforms to the horst and graben structure (Figure 7). The inter‐island area includes grabens (Figure 7B) with thicknesses increasing towards the centre and decreasing towards the margins. The base of SF1 and top of SF5 rises towards the landmasses (Figures S2 and S3) illustrating these trends.

### 4.2 Interpretation

#### 4.2.1 Outcrops

Both coralline bindstone and bafflestone to floatstone are indicative of deposition within the photic range of the water column, although they can reach into the mesophotic depth, and are not substantially affected by salinity or temperature (Adey & Macintyre, 1973; Kahng et al., 2010). Prior studies (Bosence & Pedley, 1982; Pedley, 1978, 1979) had identified *Mesophyllum* and various other Corallinales taxa associated with encrusting foraminifera and bryozoans as the main carbonate producers. Such coralline algal assemblages dominated by *Mesophyllum* and Corallinales are generally associated with shallow‐water (20–40 m) regimes (Aguirre et al., 2000; Cabioch et al., 1999; Coletti & Basso, 2020; Minnery et al., 1985). The alternation of coralline bindstone and rhodoliths suggests variation in water agitation as rhodoliths are formed in an environment with more disruption (Williams et al., 2018). The high sphericity of the rhodoliths also points to a higher energy setting (Aguirre et al., 2017; Basso, 1998; Bosence, 1983). The fragmentation of the rhodoliths within the lower parts of the section in Santi and Għajn Tuffieħa points to a relatively energetic environment, although the presence of fine grains in all localities suggests that energy is intermittent. In localities like Anchor Bay, where coralline algal bindstones are not dominant, the presence of channelised features (Figure 3E) suggests that the observed coralline algal and LBF grainstones to rudstones consist of transported material. It is unclear if the channels observed in Anchor Bay are purely subaqueous, although the absence of exposure‐related features appears to support this interpretation. One interpretation related to the occurrence of these large grain infills is that these indicate a high energy environment in shallow sublittoral settings (Bassi et al., 2017; Coletti et al., 2015). This further points to a wide energy range across the deposition of the UCL, spatially as well as temporally.

Fauna present within this upper portion of the unit, such as limpets, suggests a littoral to sublittoral position (Meadows & Campbell, 1972). This interpretation is also supported by the presence of herringbone cross‐stratification in Rdum Majjiesa, indicating intertidal conditions (Nichols, 2009). Pedley (1979, 2011), also noted the presence of stromatolites (Qammieħ Beds) and oolites (Gebel Imbark Member), both indicating tidal to peri‐tidal conditions. Stromatolites could also be subtidal but in any case would indicate restriction (Meilijson et al., 2015). This interplay with hummocky and planar cross‐stratification indicates a relatively shallow and energetic environment. The sediment mobilisation and continued deposition limited the availability of a hard substrate, which limited the ability of coralline algae to colonise. Pedley (1979) noted that the coralline bioherms would have generated a patchwork of different energy states around them, which would allow for varying energy states over a short distance until topography is filled. While there are some bioherm construction and accretion of coralline bindstone (Figure 4F), both laterally and vertically, this is mostly local. The large‐scale structure observed at the outcrop‐level (Figure 5) points to lateral distribution of material with periods marked by progradation.

With this in mind, the alternating accretion mode of the coralline algae between bindstone and rhodoliths, and the interruptions of finer grain layers between the coralline horizons (Figure 3), may be interpreted as short‐term depositional cycles, either autocyclic or allocyclic, developing by the interaction between production by the calcifying factory and accommodation space. While effects like storms and variation in runoff could disrupt coralline accumulation (Dulin et al., 2020), no storm deposits were encountered. Malta, being an isolated platform with little land area in which rainfall could accumulate, would have limited potential for local runoff generation.

The mineralogy of the UCL in the outcrop is not primary as the original constituents are dominantly high‐Mg calcite (e.g. coralline algae) or aragonite (e.g. corals). This mineral assemblage suggests that material had been recrystallised at some point post‐deposition. The absence of dolomite suggests an open system that allowed removal of the Mg. The implication of this would be that any geochemical analysis would inform of the diagenetic rather than the primary conditions (Huber et al., 2017).

The small‐scale cyclic nature of coarse‐grained rudstone/bindstone to mudstone/packstone as described here at Rdum Majjiesa, Għajn Tuffieħa and Santi can be interpreted as high‐frequency cycles that integrate into two large‐scale cycles. These lower‐order cycles govern the deposition of the UCL in the late Tortonian and early Messinian (Figure 3). It is not possible at present to determine if the higher‐order cycles are autocyclic or allocyclic. Given the enhancement of glaciation in the late Miocene (Holbourn et al., 2013), the large‐scale cycles are probably glacioisostatic of third order or higher. These large‐scale cycles are expressed differently in the various sections due to the significant variability of depositional environment across the platform with the emplacement of the UCL. Key surfaces delineating the termination of the first cycle are the occurrence of coastal and littoral deposits (Għajn Tuffieħa and Santi), serpulids (Anchor Bay) and corals (Rdum Majjiesa). This is supported by previous works (Cornée et al., 2004; Pedley, 1976, 1979, 1996), which noted that cessation of coral growth represents a major transgressive surface. No clear termination of the second cycle was found as there was no overlying deposition. Yet, the top part of this second cycle is defined by cross‐bedded grainstones with coastal characteristics (Rdum Majjiesa and Bobbyland). These observations suggest that deposition of the second cycle took place with less available accommodation space than the first cycle.

#### 4.2.2 Seismic reflection data

SU4 overlays a significant unconformity and exhibits a significant shift in seismic facies. Based on existing knowledge of the configuration of deposits on the Maltese platform (Gatt, 2007; Max et al., 1993; Micallef et al., 2011; Osler & Algan, 1999), the deposits of SU4 are probably Plio‐Quaternary in age and the base of this unit is an unconformity related to the MSC (Messinian erosional surface) or the subsequent Zanclean megaflood (Garcia‐Castellanos et al., 2020). The underlying SU3 would therefore be part of the UCL. The unit is truncated, and its thickness decreases to 0 ms to the south‐west (Figure S4).

The lateral continuation from SF2 to SF4 (Figure 6C) observed in both SU3 and SU2 points to similar depositional systems in both units. The morphology observed by SF2 is consistent with the seismic character of a carbonate buildup (Burgess et al., 2013). Its position relative to the islands also suggests that this structure could be the lateral continuation of the UCL main bioherms observed in the outcrops (Pedley, 1976, 1979). As such SF2 is interpreted as a bioherm, which would suggest that both SU3 and SU2 combined are the equivalent of the UCL, with each unit representing a depositional cycle. The maximum thickness of this combined unit is *ca* 100 ms, which is comparable to the maximum thickness of *ca* 80 m reported by Cornée et al. (2004) for the UCL. These bioherms exhibit the convex margins that would be expected from the less lithified and wave resistant C‐type factory buildups (Schlager, 2005). By extension, SF3 would probably mark large‐scale clinoforms spilling off the main bioherm, as observed onshore (Figure 5), and SF4 would be a protected environment behind the outer bioherms. The onlap contact of SF4 suggests that this facies was deposited in the later part of the depositional cycle, filling up available accommodation space. Schlager (2005) defined the C‐type carbonate factory as one dominated by material from a biological source rich in heterotrophic organisms, larger benthic foraminifera and coralline algae. This type of factory differs from the type of carbonate production in a tropical carbonate platform (also called T‐type factory). Whereas in the T‐type factory carbonate production mainly consists of a rigid framework (e.g. a coral‐reef), the C‐type factory mainly produces sand‐to‐granule sized skeletal grains. A coralline algae dominated C‐Type factory, such as the UCL, would export significant amounts of grains to the surrounding environment. The position and main production zone might move laterally in response to a change in sea level and accumulation would respond strongly to wave activity. The bioherms found in C‐type factories are not as ridged as reefs in T‐type factories. Sediment lithification can occur in marine environments but is usually relegated only locally to the bioherms or is related to different types of discontinuity surfaces (Nelson et al., 2003). This in turn would fit some of the strong but non‐continuous reflections observed within the UCL (Figures 6 and 7).

The underlying horst and graben structure of the Maltese Archipelago (Dart et al., 1993) predated the deposition of the UCL, and the offshore deposit conforms to the pre‐existing structure (Figure 7). This situation may suggest that the deposits observed in the seismic reflection data were deposited in a lower topographic position, with possible overspill from Malta and Gozo observed on the margins of the grabens (Figure 7C). As such, SF4 probably has a strong lateral transport component from the main production zone as it shifted with increasing relative sea level.

Finally, SU1 contains within it the pre‐UCL units that include the Globigerina Limestone, Blue Clay and Greensand. Given that the thickness of the GS is only a few metres (Pedley, 1978), it is probably not observed in the seismic data and/or incorporated in the Base UCL horizon. Therefore, the upper part of SU 1 is considered to be the Blue Clay Formation.

## 5 DISCUSSION

**FIGURE 8:**
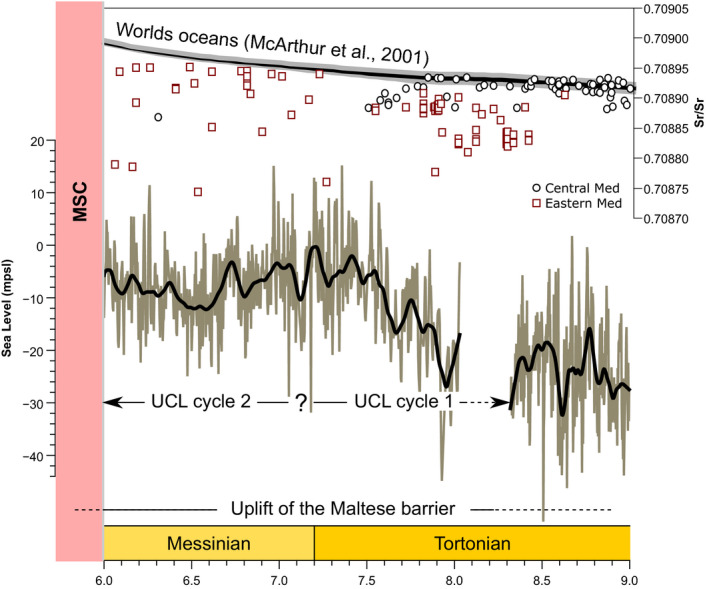
Temporal context of the deposition of the UCL, Sr isotope record of the Eastern (Flecker & Ellam, 2006; Schildgen et al., 2014) and Central (Kocsis et al., 2008; Schildgen et al., 2014; Sprovieri et al., 2004) Mediterranean relative to the coeval oceanic reference (McArthur et al., 2001). Global sea level (Miller et al., 2020) and proposed relationship to the deposition of the UCL cycles

The most recent model of global sea‐level change in the late Miocene (Miller et al., 2020) depicts a base level rise of *ca* 20 m in the late Tortonian (*ca* 8–7.5 Ma, Figure 8). After this rise, mean sea level remained relatively static until the MSC. Baldassini and Di Stefano (2017) inferred that the age of the base of the UCL is at *ca* 8 Ma and the top of it lies within the MNN11 nanofossil zone. Dolomitisation appears to have occurred wherever such interaction took place (de Lange & Krijgsman, 2010; Manzi et al., 2011; Sabino et al., 2020). This relationship appears to be the result of vigorous reflux circulation with extensive microbial activity. However, no dolomite was detected in the mineralogical analysis, suggesting minimal interaction of hypersaline MSC fluids with any of the Maltese deposits. This limits the age of the top of the UCL to no more *ca* 6 Ma. Some collapse breccia with local gypsum cementation can be found in a few localities (Pedley, 2011) and could be either MSC products or local restriction due to subaerial emergence. The emplacement of a shallow‐water unit/mass atop a deepwater unit such as the Blue Clay Formation (Abels et al., 2005), while sea level was rising, already suggests a major uplift of the Maltese Plateau during the late Miocene. By examining the ^87^Sr/^86^Sr_carbonate_ record of the Eastern Mediterranean at the time (Figure 8; Flecker & Ellam, 2006; Schildgen et al., 2014), it is seen to exhibit significantly lower values when compared with the coeval oceanic reference value (McArthur et al., 2001). Lower values are also observed in the central Mediterranean (Kocsis et al., 2008; Schildgen et al., 2014; Sprovieri et al., 2004), but these are no lower than 0.70887, while the samples in the Eastern Mediterranean can be lower than 0.70880. Notably, Cornacchia et al. (2017), working in the Majella Mountain (Eastern Mediterranean domain, proto Adriatic) observed lower ^87^Sr/^86^Sr ratios on a comparable timeframe, interpreted as increased local overprint with regional restriction.

These values are still higher than the departure observed during the MSC (Flecker et al., 2002; Müller & Mueller, 1991; Roveri et al., 2014b), but the trend is in the same direction. Overall the ^87^Sr/^86^Sr ratio of the ocean is governed by the balance between continental weathering and hydrothermal supply from mid‐ocean ridges (Jones et al., 1994). However, in a restricted basin, the contribution from terrestrial sources may overprint the oceanic signal. Together, these two lines of evidence suggest a restricted exchange between the Eastern and Western Mediterranean due to uplift of the terrain between Sicily and Tunisia. This uplift appears to have been driven by the aftermath of the collision between the African margin and the Sicilian Block (Jongsma et al., 1985), with a total uplift of >500 m during the Tortonian to the earliest Pliocene (Yellin‐Dror et al., 1997). No clear indication of such movement is observed in the seismic data available for this study. Palaeodepth estimation at the top of the Blue Clay Formation (Serravallian) is estimated at 150–200 m (Digeronimo et al., 1981; Jacobs et al., 1996), while coastal to littoral deposits were identified in the upper part of the UCL (upper Messinian). Considering that the total thickness of the UCL deposits is <30 m, the filling of accommodation space alone could not account for these observations. The total accommodation is also offset but *ca* 25 m of sea‐level rise during deposition of the UCL (Miller et al., 2020, Figure 8) suggests an uplift of at least 150 m in Malta.

The emplacement of two cycles of shallow‐water deposits in the UCL formation, terminating near sea level, indicates that accommodation space was available for the establishment of two depositional cycles and topography fill by a carbonate factory. The fact that a C‐type factory is dominant in Malta at this time, while in other regions of the Mediterranean T‐type factories are still dominant (Follows, 1992; Reolid et al., 2014), does suggest that the conditions in this region were less favourable. The wide and shallow water mass extended due to the uplift could result in higher salinity or a strong temperature seasonal amplitude, both could inhibit the development of a T‐type factory favouring the emplacement of a C‐type factory. Each cycle (Figure 6) begins with the local establishment of bioherms, which grow vertically with a lateral component (aggradation–progradation), followed by a phase of lateral growth (progradation) and finally a stage dominated by filling of accommodation space (aggradation). The initial two phases would be comparable to the initial shallowing upwards phase observed in the outcrops, with the aggradational phases being comparable to the deepening upwards phase. The available timeframe of emplacement of the UCL (from *ca* 8 to *ca* 6 Ma), and the known eustatic sea‐level patterns (Miller et al., 2020), suggest that the depositional cycles are of the order of 1 Ma each, likely governed by 1.2 Ma eccentricity modulated astronomical frequencies, and represent two third order cycles (Boulila et al., 2012). This overall trend of fill points to limited accommodation space generation. Some accommodation was still generated by coeval sea‐level rise (Figure 8) and local tectonics. The latter by formation of local basins as indicated by the fault‐bound nature of the sedimentary bodies observed in seismic data (Figure 7). The overall stress field at the time would have been a compressional one along a north–south vector due to collision. This compression generated an extensional regime along an east–west vector in the late Miocene and early Pliocene (Civile et al., 2008). This trend was further enhanced by coeval rifting along this direction (Belguith et al., 2013; Corti et al., 2006). While this process would have generated local basins, the overall effect of this compressional regime would have been to magnify the total uplift of the barrier.

## 6 CONCLUSIONS

Over the late Miocene, the area around the Maltese Archipelago has experienced a significant uplift. The uplift raised the Maltese Islands to a water depth that allowed the emplacement of a shallow‐water carbonate unit in the form of the UCL Formation. This unit was deposited during two depositional cycles, with both cycles characterised by a strong tendency towards filling the available accommodation space typical of C‐type carbonate factories. The mode of emplacement and overall geometry suggests a very shallow position of the Malta Plateau, conforming to an existing tectonic structure already present in the late Miocene. This uplift appears to have played a role in restricting connectivity of the water masses between the east and the west Mediterranean prior to the onset of the MSC and could have played an important role in triggering halite deposition in the larger Eastern Basin prior to salt deposition in the Western Basin. This uplift would have restricted flow to the Eastern Basin at a time when the Western Basin was still relatively well‐connected to the Atlantic Ocean and probably played a pivotal role in the observed differences in deposits between the two basins during the MSC.

## Supporting information


Figure S1
Figure S2
Figure S3

## Data Availability

Seismic data included in this study were provided by Infrastructure Malta and subject to third party restrictions.

## References

[dep2138-bib-0001] Abels, H.A., Hilgen, F.J., Krijgsman, W., Kruk, R.W., Raffi, I., Turco, E. et al. (2005) Long‐period orbital control on middle Miocene global cooling: Integrated stratigraphy and astronomical tuning of the Blue Clay Formation on Malta. Paleoceanography, 20, PA4012. 10.1029/2004PA001129

[dep2138-bib-0002] Adam, J., Reuther, C.‐D., Grasso, M. & Torelli, L. (2000) Active fault kinematics and crustal stresses along the Ionian margin of southeastern Sicily. Tectonophysics, 326, 217–239. 10.1016/S0040-1951(00)00141-4

[dep2138-bib-0003] Adey, W.H. & Macintyre, I.G. (1973) Crustose coralline algae: A re‐evaluation in the geological sciences. Geological Society of America Bulletin, 84, 883. 10.1130/0016-7606(1973)84<883:CCAARI>2.0.CO;2

[dep2138-bib-0004] Aguirre, J., Braga, J.C. & Bassi, D. 2017. Rhodoliths and rhodolith beds in the rock record. In: Riosmena‐Rodríguez, R. (Ed.), Rhodolith/Maërl beds: A global perspective, Springer, Coastal Research Library, vol. 15, pp. 105–138.10.1007/978‐3‐319‐29315‐8_5

[dep2138-bib-0005] Aguirre, J., Riding, R. & Braga, J.C. (2000) Late Cretaceous incident light reduction: Evidence from benthic algae. Lethaia, 33, 205–213. 10.1080/00241160025100062

[dep2138-bib-0006] Baldassini, N. & Di Stefano, A. (2017) Stratigraphic features of the Maltese Archipelago: A synthesis. Natural Hazards, 86, 203–231. 10.1007/s11069-016-2334-9

[dep2138-bib-0007] Bassi, D., Simone, L. & Nebelsick, J.H. (2017) Re‐sedimented rhodoliths in channelized depositional systems. In: Riosmena‐Rodríguez, R., Nelson, W. & Aguirre, J. (Eds.) Rhodolith/Maërl beds: A global perspective, pp. Switzerland: Springer, Coastal Research Library 15, 139–167. 10.1007/978-3-319-29315-8_6

[dep2138-bib-0008] Basso, D. (1998) Deep rhodolith distribution in the Pontian Islands, Italy: A model for the paleoecology of a temperate sea. Palaeogeography, Palaeoclimatology, Palaeoecology, 137, 173–187. 10.1016/S0031-0182(97)00099-0

[dep2138-bib-0009] Belguith, Y., Geoffroy, L., Mourgues, R. & Rigane, A. (2013) Analogue modelling of Late Miocene‐Early quaternary continental crustal extension in the Tunisia‐Sicily Channel area. Tectonophysics, 608, 576–585. 10.1016/j.tecto.2013.08.023

[dep2138-bib-0010] Ben‐Avraham, Z., Nur, A. & Giuseppe, C. (1987) Active transcurrent fault system along the north African passive margin. Tectonophysics, 141, 249–260. 10.1016/0040-1951(87)90189-2

[dep2138-bib-0011] Blanc, P.‐L. (2000) Of sills and straits: A quantitative assessment of the Messinian Salinity Crisis. Deep Sea Research Part I: Oceanographic Research Papers, 47, 1429–1460. 10.1016/S0967-0637(99)00113-2

[dep2138-bib-0012] Blanc, P.L. (2006) Improved modelling of the Messinian salinity crisis and conceptual implications. Palaeogeography, Palaeoclimatology, Palaeoecology, 238, 349–372. 10.1016/j.palaeo.2006.03.033

[dep2138-bib-0013] Borrelli, M., Perri, E., Critelli, S. & Gindre‐Chanu, L. (2020) The onset of the Messinian Salinity Crisis in the central Mediterranean recorded by pre‐salt carbonate/evaporite deposition. Sedimentology. 10.1111/sed.12824

[dep2138-bib-0014] Bosence, D.W.J. (1983) Description and classification of Rhodoliths (Rhodoids, Rhodolites). In: Peryt, T.M. (Ed.) Coated grains. Berlin Heidelberg: Springer, pp. 217–224. 10.1007/978-3-642-68869-0_19

[dep2138-bib-0015] Bosence, D.W.J. & Pedley, H.M. (1982) Sedimentology and palaeoecology of a Miocene coralline algal biostrome from the Maltese Islands. Palaeogeography, Palaeoclimatology, Palaeoecology, 38, 9–43. 10.1016/0031-0182(82)90062-1

[dep2138-bib-0016] Boulila, S., Galbrun, B., Laskar, J. & Pälike, H. (2012) A ~9 Myr cycle in Cenozoic δ13C record and long‐term orbital eccentricity modulation: Is there a link? Earth and Planetary Science Letters, 317‐318, 273–281. 10.1016/j.epsl.2011.11.017

[dep2138-bib-0017] Buchbinder, B. (1996) Miocene carbonates of the Eastern Mediterranean, the Red Sea and the Mesopotamian Basin: Geodynamic and eustatic controls. In: Franseen, E.K., Esteban, M., Ward, W.C. & Rouchy, J.‐M. (Eds.), Models for carbonate stratigraphy from Miocene Reef complexes of Mediterranean regions. *SEPM Concepts in Sedimentology and Paleontology*, vol. 5, 333–345.10.2110/csp.96.01.0317

[dep2138-bib-0018] Burgess, P.M., Winefield, P., Minzoni, M. & Elders, C. (2013) Methods for identification of isolated carbonate buildups from seismic reflection data. American Association of Petroleum Geologists Bulletin, 97, 1071–1098. 10.1306/12051212011

[dep2138-bib-0019] Cabioch, G., Montaggioni, L.F., Faure, G. & Ribaud‐Laurenti, A. (1999) Reef coralgal assemblages as recorders of paleobathymetry and sea level changes in the Indo‐Pacific province. Quaternary Science Reviews, 18, 1681–1695. 10.1016/S0277-3791(99)00014-1

[dep2138-bib-0020] Catanzariti, R. & Gatt, M. (2014) Calcareous nannofossil biostratigraphy from the middle/late Miocene of Malta and Gozo (Central Mediterranean). Stratigraphy, 11, 303–336.

[dep2138-bib-0021] Civile, D., Lodolo, E., Tortorici, L., Lanzafame, G. & Brancolini, G. (2008) Relationships between magmatism and tectonics in a continental rift: The Pantelleria Island region (Sicily Channel, Italy). Marine Geology, 251, 32–46. 10.1016/j.margeo.2008.01.009

[dep2138-bib-0022] Coletti, G. & Basso, D. (2020) Coralline algae as depth indicators in the Miocene carbonates of the Eratosthenes Seamount (ODP Leg 160, Hole 966F). Geobios, 60, 29–46. 10.1016/j.geobios.2020.03.005

[dep2138-bib-0023] Coletti, G., Basso, D., Betzler, C., Robertson, A.H.F., Bosio, G., El Kateb, A. et al. (2019) Environmental evolution and geological significance of the Miocene carbonates of the Eratosthenes Seamount (ODP Leg 160). Palaeogeography, Palaeoclimatology, Palaeoecology, 530, 217–235. 10.1016/j.palaeo.2019.05.009

[dep2138-bib-0024] Coletti, G., Basso, D., Frixa, A. & Corselli, C. (2015) Transported Rhodoliths witness the lost carbonate factory: A case history from the Miocene pietra da cantoni limestone (NW Italy). Rivista Italiana di Paleontologia e Stratigrafia, 121, 345–368. 10.13130/2039-4942/6522

[dep2138-bib-0025] Cornacchia, I., Andersson, P., Agostini, S., Brandano, M. & Di Bella, L. (2017) Strontium stratigraphy of the upper Miocene Lithothamnion Limestone in the Majella Mountain, central Italy, and its palaeoenvironmental implications. Lethaia, 50, 561–575. 10.1111/let.12213

[dep2138-bib-0026] Cornée, J.J., Saint Martin, J.P., Conesa, G., Münch, P., André, J.P., Saint Martin, S. et al. (2004) Correlations and sequence stratigraphic model for Messinian carbonate platforms of the western and central Mediterranean. International Journal of Earth Sciences, 93, 621–633. 10.1007/s00531-004-0400-0

[dep2138-bib-0027] Corti, G., Cuffaro, M., Doglioni, C., Innocenti, F. & Manetti, P. (2006) Coexisting geodynamic processes in the Sicily Channel. Special Papers – Geological Society of America, 409, 83–96. 10.1130/2006.2409(05)

[dep2138-bib-0028] Dart, C.J., Bosence, W.J. & McClay, K.R. (1993) Stratigraphy and structure of the Maltese graben system. Journal of the Geological Society of London, 150, 1153–1166. 10.1144/gsjgs.150.6.1153

[dep2138-bib-0029] de Lange, G.J. & Krijgsman, W. (2010) Messinian salinity crisis: A novel unifying shallow gypsum/deep dolomite formation mechanism. Marine Geology, 275, 273–277. 10.1016/j.margeo.2010.05.003

[dep2138-bib-0030] Digeronimo, I., Grasso, M. & Pedley, H.M. (1981) Palaeoenvironment and palaeogeography of Miocene marls from southeast Sicily and the Maltese Islands. Palaeogeography, Palaeoclimatology, Palaeoecology, 34, 173–189. 10.1016/0031-0182(81)90063-8

[dep2138-bib-0031] Dulin, T., Avnaim‐Katav, S., Sisma‐Ventura, G., Bialik, O.M. & Angel, D.L. (2020) Rhodolith beds along the southeastern Mediterranean inner shelf: Implications for past depositional environments. Journal of Marine Systems, 201, 103241. 10.1016/j.jmarsys.2019.103241

[dep2138-bib-0032] Dunham, R.J. 1962. Classification of carbonate rocks according to depositional textures. In: Ham, W.E. (Ed.), Classification of carbonate rocks – A symposium. *AAPG Memoir* 1, 108–121.

[dep2138-bib-0033] Embry, A.F. & Klovan, J.E. (1971) A Late Devonian reef tract on northeastern Banks Island, NWT. Bulletin of Canadian Petroleum Geology, 19, 730–781.

[dep2138-bib-0034] Esteban, M. (1979) Significance of the upper Miocene coral reefs of the Western Mediterranean. Palaeogeography, Palaeoclimatology, Palaeoecology, 29, 169–188. 10.1016/0031-0182(79)90080-4

[dep2138-bib-0035] Flecker, R., De Villiers, S. & Ellam, R.M. (2002) Modelling the effect of evaporation on the salinity‐^87^Sr/^86^Sr relationship in modern and ancient marginal‐marine systems: The Mediterranean Messinian salinity crisis. Earth and Planetary Science Letters, 203, 221–233. 10.1016/S0012-821X(02)00848-8

[dep2138-bib-0036] Flecker, R. & Ellam, R.M.M. (2006) Identifying Late Miocene episodes of connection and isolation in the Mediterranean‐Paratethyan realm using Sr isotopes. Sedimentary Geology, 188–189, 189–203. 10.1016/j.sedgeo.2006.03.005

[dep2138-bib-0037] Follows, E.J. (1992) Patterns of reef sedimentation and diagenesis in the Miocene of Cyprus. Sedimentary Geology, 79, 225–253. 10.1016/0037-0738(92)90013-H

[dep2138-bib-0038] Garcia‐Castellanos, D., Micallef, A., Estrada, F., Camerlenghi, A., Ercilla, G., Periáñez, R. et al. (2020) The Zanclean megaflood of the Mediterranean – Searching for independent evidence. Earth‐Science Reviews, 201, 103061. 10.1016/j.earscirev.2019.103061

[dep2138-bib-0039] Gardiner, W., Grasso, M. & Sedgeley, D. (1995) Plio‐pleistocene fault movement as evidence for mega‐block kinematics within the Hyblean—Malta Plateau, Central Mediterranean. Journal of Geodynamics, 19, 35–51. 10.1016/0264-3707(94)00006-9

[dep2138-bib-0040] Gatt, P.A. (2007) Controls on Plio‐Quaternary foreland sedimentation in the region of the Maltese Islands. Bollettino della Società Geologica Italiana, 126, 119–129.

[dep2138-bib-0041] Gatt, P.A. & Gluyas, J.G. (2012) Climatic controls on facies in Palaeogene Mediterranean subtropical carbonate platforms. Petroleum Geoscience, 18, 355–367. 10.1144/1354-079311-032

[dep2138-bib-0042] Gutscher, M.‐A., Dominguez, S., de Lepinay, B.M., Pinheiro, L., Gallais, F., Babonneau, N. et al. (2016) Tectonic expression of an active slab tear from high‐resolution seismic and bathymetric data offshore Sicily (Ionian Sea). Tectonics, 35, 39–54. 10.1002/2015TC003898

[dep2138-bib-0043] Haq, B., Gorini, C., Baur, J., Moneron, J. & Rubino, J.‐L. (2020) Deep Mediterranean’s Messinian evaporite giant: How much salt? Global and Planetary Change, 184, 103052. 10.1016/j.gloplacha.2019.103052

[dep2138-bib-0044] Holbourn, A., Kuhnt, W., Clemens, S., Prell, W. & Andersen, N. (2013) Middle to late Miocene stepwise climate cooling: Evidence from a high‐resolution deep water isotope curve spanning 8 million years. Paleoceanography, 28, 688–699. 10.1002/2013PA002538

[dep2138-bib-0045] Hsü, K., Ryan, W. & Cita, M. (1973) Late Miocene desiccation of the Mediterranean. Nature, 242, 240–244.

[dep2138-bib-0046] Huber, C., Druhan, J.L. & Fantle, M.S. (2017) Perspectives on geochemical proxies: The impact of model and parameter selection on the quantification of carbonate recrystallisation rates. Geochimica et Cosmochimica Acta, 217, 171–192. 10.1016/j.gca.2017.08.023

[dep2138-bib-0047] Jacobs, E., Weissert, H., Shields, G. & Stille, P. (1996) The monterey event in the Mediterranean: A record from shelf sediments of Malta. Paleoceanography, 11, 717–728. 10.1029/96PA02230

[dep2138-bib-0048] Jones, C.E., Jenkyns, H.C., Coe, A.L. & Stephen, H.P. (1994) Strontium isotopic variations in Jurassic and Cretaceous seawater. Geochimica et Cosmochimica Acta, 58, 3061–3074. 10.1016/0016-7037(94)90179-1

[dep2138-bib-0049] Jongsma, D., van Hinte, J.E. & Woodside, J.M. (1985) Geologic structure and neotectonics of the North African continental margin south of Sicily. Marine and Petroleum Geology, 2, 156–179. 10.1016/0264-8172(85)90005-4

[dep2138-bib-0050] Kahng, S.E., Garcia‐Sais, J.R., Spalding, H.L., Brokovich, E., Wagner, D., Weil, E. et al. (2010) Community ecology of mesophotic coral reef ecosystems. Coral Reefs, 29, 255–275. 10.1007/s00338-010-0593-6

[dep2138-bib-0051] Kocsis, L., Vennemann, T.W., Fontignie, D., Baumgartner, C., Montanari, A. & Jelen, B. (2008) Oceanographic and climatic evolution of the Miocene Mediterranean deduced from Nd, Sr, C, and O isotope compositions of marine fossils and sediments. Paleoceanography, 23, 1–20. 10.1029/2007PA001540

[dep2138-bib-0052] Kontakiotis, G., Besiou, E., Antonarakou, A., Zarkogiannis, S.D., Kostis, A., Mortyn, P.G. et al. (2019) Decoding sea surface and paleoclimate conditions in the eastern Mediterranean over the Tortonian‐Messinian Transition. Palaeogeography, Palaeoclimatology, Palaeoecology, 534, 109312. 10.1016/j.palaeo.2019.109312

[dep2138-bib-0053] Kouwenhoven, T.J., Morigi, C., Negri, A., Giunta, S., Krijgsman, W. & Rouchy, J.M. (2006) Paleoenvironmental evolution of the eastern Mediterranean during the Messinian: Constraints from integrated microfossil data of the Pissouri Basin (Cyprus). Marine Micropaleontology, 60, 17–44. 10.1016/j.marmicro.2006.02.005

[dep2138-bib-0054] Krijgsman, W., Blanc‐Valleron, M.M., Flecker, R., Hilgen, F.J., Kouwenhoven, T.J., Merle, D. et al. (2002) The onset of the Messinian salinity crisis in the Eastern Mediterranean (Pissouri Basin, Cyprus). Earth and Planetary Science Letters, 194, 299–310. 10.1016/S0012-821X(01)00574-X

[dep2138-bib-0055] Manzi, V., Lugli, S., Roveri, M., Schreiber, B.C. & Gennari, R. (2011) The Messinian “Calcare di Base” (Sicily, Italy) revisited. Bulletin of Geological Society of America, 123, 347–370. 10.1130/B30262.1

[dep2138-bib-0056] Max, M.D., Kristensen, E. & Michelozzi, E. (1993) Small scale Plio‐Quaternary sequence stratigraphy and shallow geology of the west‐central Malta Plateau. In: Max, M.D. and Colantoni, P. (Eds.) UNESCO technical reports in marine science. Urbino: International Oceanographic Data and Information Exchange, pp. 117–122.

[dep2138-bib-0057] McArthur, A.J.M., Howarth, R.J., Bailey, T.R., Mcarthur, J.M., Howarth, R.J. & Bailey, T.R. (2001) Strontium isotope stratigraphy: LOWESS version 3: Best fit to the marine Sr‐isotope curve for 0–509 Ma and accompanying look‐up table for deriving numerical age. The Journal of Geology, 109, 155–170. 10.1086/319243

[dep2138-bib-0058] Meadows, P.S. & Campbell, J.I. (1972) Habitat selection by aquatic invertebrates. In: Russell, F.S. and Yonge, M. (Eds.) Advances in marine biology, vol. 10. London: Academic Press, pp. 271–382. 10.1016/S0065-2881(08)60418-6

[dep2138-bib-0059] Meijer, P.T. (2006) A box model of the blocked‐outflow scenario for the Messinian salinity crisis. Earth and Planetary Science Letters, 248, 486–494. 10.1016/j.epsl.2006.06.013

[dep2138-bib-0060] Meilijson, A., Bialik, O.M. & Benjamini, C. (2015) Stromatolitic biotic systems in the mid‐Triassic of Israel – A product of stress on an epicontinental margin. Palaeogeography, Palaeoclimatology, Palaeoecology, 440, 696–711, 10.1016/j.palaeo.2015.09.030

[dep2138-bib-0061] Meilijson, A., Hilgen, F., Sepúlveda, J., Steinberg, J., Fairbank, V., Flecker, R. et al. (2019) Chronology with a pinch of salt: Integrated stratigraphy of Messinian evaporites in the deep Eastern Mediterranean reveals long‐lasting halite deposition during Atlantic connectivity. Earth‐Science Reviews, 194, 374–398. 10.1016/j.earscirev.2019.05.011

[dep2138-bib-0062] Meilijson, A., Steinberg, J., Hilgen, F., Bialik, O.M., Waldmann, N.D. & Makovsky, Y. (2018) Deep‐basin evidence resolves a 50‐year‐old debate and demonstrates synchronous onset of Messinian evaporite deposition in a non‐desiccated Mediterranean. Geology, 46, 10.1130/G39868

[dep2138-bib-0063] Micallef, A., Berndt, C. & Debono, G. (2011) Fluid flow systems of the Malta Plateau, Central Mediterranean Sea. Marine Geology, 284, 74–85. 10.1016/j.margeo.2011.03.009

[dep2138-bib-0064] Micallef, A., Georgiopoulou, A., Mountjoy, J., Huvenne, V.A.I., Iacono, C.L., Le Bas, T. et al. (2016) Outer shelf seafloor geomorphology along a carbonate escarpment: The eastern Malta Plateau, Mediterranean Sea. Continental Shelf Research, 131, 12–27. 10.1016/j.csr.2016.11.002

[dep2138-bib-0065] Miller, K.G., Browning, J.V., Schmelz, W.J., Kopp, R.E., Mountain, G.S. & Wright, J.D. (2020) Cenozoic sea‐level and cryospheric evolution from deep‐sea geochemical and continental margin records. Science Advances, 6, eaaz1346. 10.1126/sciadv.aaz1346 32440543 PMC7228749

[dep2138-bib-0066] Minnery, G.A., Rezak, R. & Bright, T.J. (1985) Depth zonation and growth form of Crustose coralline algae: Flower garden banks, Northwestern Gulf of Mexico. In: Toomey, D.F. and Nitecki, M.H. (Eds.) Paleoalgology. Berlin, Heidelberg: Springer, pp. 237–246. 10.1007/978-3-642-70355-3_18

[dep2138-bib-0067] Moissette, P., Cornée, J.J., Antonarakou, A., Kontakiotis, G., Drinia, H., Koskeridou, E. et al. (2018) Palaeoenvironmental changes at the Tortonian/Messinian boundary: A deep‐sea sedimentary record of the eastern Mediterranean Sea. Palaeogeography, Palaeoclimatology, Palaeoecology, 505, 217–233. 10.1016/j.palaeo.2018.05.046

[dep2138-bib-0068] Müller, D.W. & Mueller, P.A. (1991) Origin and age of the Mediterranean Messinian evaporites: Implications from Sr isotopes. Earth and Planetary Science Letters, 107, 1–12. 10.1016/0012-821X(91)90039-K

[dep2138-bib-0069] Nelson, C.S., Winefield, P.R., Hood, S.D., Caron, V., Pallentin, A. & Kamp, P.J.J. (2003) Pliocene Te Aute limestones, New Zealand: Expanding concepts for cool‐water shelf carbonates. New Zealand Journal of Geology and Geophysics, 46, 407–424. 10.1080/00288306.2003.9515017

[dep2138-bib-0070] Nichols, G. (2009) Sedimentology and stratigraphy. Oxford: Wiley‐Blackwell.

[dep2138-bib-0071] Osler, J.C. & Algan, O. (1999) A high resolution seismic sequence analysis of the Malta Plateau. La Spezia, Italy: NATO, Report No. SR‐311‐UU.

[dep2138-bib-0072] Pedley, H.M. (1976) A palaeoecological study of the Upper Coralline Limestone, Terebratula‐Aphelesia bed (Miocene, Malta) based on bryozoan growth‐form studies and brachiopod distributions. Palaeogeography, Palaeoclimatology, Palaeoecology, 20, 209–234. 10.1016/0031-0182(76)90003-1

[dep2138-bib-0073] Pedley, H.M. (1978) A new lithostratigraphical and palaeoenvironmental interpretation Forthe Coralli ne limestoneformations (Miocene) of the Maltese Islands. Overseas Geology and Mineral Resources, 54, 1–17.

[dep2138-bib-0074] Pedley, H.M. (1979) Miocene bioherms and associated structures in the Upper Coralline limestone of the Maltese Islands: Their lithification and palaeoenvironment. Sedimentology, 26, 577–591. 10.1111/j.1365-3091.1979.tb00930.x

[dep2138-bib-0075] Pedley, H.M. (1996) Miocene reef facies of the Pelagian region (central Mediterranean). In: Franseen, E.K., Esteban, M., Ward, W.C. & Rouchy, J.‐M. (Eds.) Models for carbonate stratigraphy from Miocene reef complexes of Mediterranean regions. *SEPM (Society for Sedimentary Geology)*, Tulsa, Oklahoma: SEPM Concepts in Sedimentology and Paleontology 5, 247–259.

[dep2138-bib-0076] Pedley, H.M. (2011) The Calabrian Stage, Pleistocene highstand in Malta: A new marker for unravelling the Late Neogene and Quaternary history of the islands. Journal of the Geological Society of London, 168, 913–926. 10.1144/0016-76492010-080

[dep2138-bib-0077] Reolid, J., Betzler, C., Braga, J.C., Martín, J.M., Lindhorst, S. & Reijmer, J.J.G. (2014) Reef slope geometries and facies distribution: controlling factors (Messinian, SE Spain). Facies, 60, 737–753. 10.1007/s10347-014-0406-4

[dep2138-bib-0078] Reuther, C.‐D., Ben‐Avraham, Z. & Grasso, M. (1993) Origin and role of major strike‐slip transfers during plate collision in the central Mediterranean. Terra Nova, 5, 249–257. 10.1111/j.1365-3121.1993.tb00256.x

[dep2138-bib-0079] Roveri, M., Flecker, R., Krijgsman, W., Lofi, J., Lugli, S., Manzi, V. et al. (2014a) The Messinian salinity crisis: Past and future of a great challenge for marine sciences. Marine Geology, 352, 25–58. 10.1016/j.margeo.2014.02.002

[dep2138-bib-0080] Roveri, M., Lugli, S., Manzi, V., Gennari, R. & Schreiber, B.C. (2014b) High‐resolution strontium isotope stratigraphy of the Messinian deep mediterranean basins: Implications for marginal to central basins correlation. Marine Geology, 349, 113–125. 10.1016/j.margeo.2014.01.002

[dep2138-bib-0081] Ryan, W.B.F. (2008) Modeling the magnitude and timing of evaporative drawdown during the Messinian salinity crisis. Stratigraphy, 5, 227–243.

[dep2138-bib-0082] Sabino, M., Dela Pierre, F., Natalicchio, M., Birgel, D., Gier, S., Peckmann, J. (2020) The response of water column and sedimentary environments to the advent of the Messinian salinity crisis: insights from an onshore deep‐water section (Govone, NW Italy). Geological Magazine, 1–17. 10.1017/s0016756820000874

[dep2138-bib-0083] Schildgen, T.F., Cosentino, D., Frijia, G., Castorina, F., Dudas, F.O., Iadanza, A. et al. (2014) Sea level and climate forcing of the Sr isotope composition of late Miocene Mediterranean marine basins. Geochemistry, Geophysics, Geosystems, 2964–2983. 10.1002/2014GC005332

[dep2138-bib-0084] Schlager, W. (2005) Carbonate sedimentology and sequence stratigraphy. SEPM concepts in sedimentology and paleontology, vol. 8, Tulsa, OK. 10.2110/csp.05.08

[dep2138-bib-0085] Spatola, D., del Moral‐Erencia, J.D., Micallef, A., Camerlenghi, A., Garcia‐Castellanos, D., Gupta, S. et al. (2020) A single‐stage megaflood at the termination of the Messinian salinity crisis: Geophysical and modelling evidence from the eastern Mediterranean Basin. Marine Geology, 430, 106337. 10.1016/j.margeo.2020.106337

[dep2138-bib-0086] Sprovieri, M., Bonanno, A., Barbieri, M., Bellanca, A., Neri, R., Patti, B. et al. (2004) ^87^Sr/^86^Sr variation in Tortonian Mediterranean sediments: A record of Milankovitch cyclicity. In: D’argenio, B., Fisher, A.G., Premoli Silva, I., Weissert, H. & Ferreri, V.(Eds.) Cyclostratigraphy: Approaches and case histories. *Society for Sedimentary Geology*, 81, 17–26. 10.2110/pec.04.81.0017

[dep2138-bib-0087] Williams, S., Halfar, J., Zack, T., Hetzinger, S., Blicher, M. & Juul‐Pedersen, T. (2018) Comparison of climate signals obtained from encrusting and free‐living rhodolith coralline algae. Chemical Geology, 476, 418–428. 10.1016/j.chemgeo.2017.11.038

[dep2138-bib-0088] Yellin‐Dror, A., Grasso, M., Ben‐Avraham, Z. & Tibor, G. (1997) The subsidence history of the northern Hyblean plateau margin, southeastern Sicily. Tectonophysics, 282, 277–289. 10.1016/S0040-1951(97)00228-X

