# Supplementary material for: Architecture and sequence stratigraphy of the Upper Coralline Limestone formation, Malta—Implications for Eastern Mediterranean restriction prior to the Messinian Salinity Crisis
Source: Depos Rec. 2021 Mar 2;7(2):256–70. doi: 10.1002/dep2.138 (PMC8359163; doi:10.1002/dep2.138)
Supplement: Supplementary file 1 — Figure S1 Figure S2 Figure S3 [file DEP2-7-256-s001.pdf]

Architecture and sequence stratigraphy of the Upper Coralline Algae Formation, Malta – implications for eastern Mediterranean restriction prior to the Messinian Salinity Crisis

Supplementary Figures

Figure S1

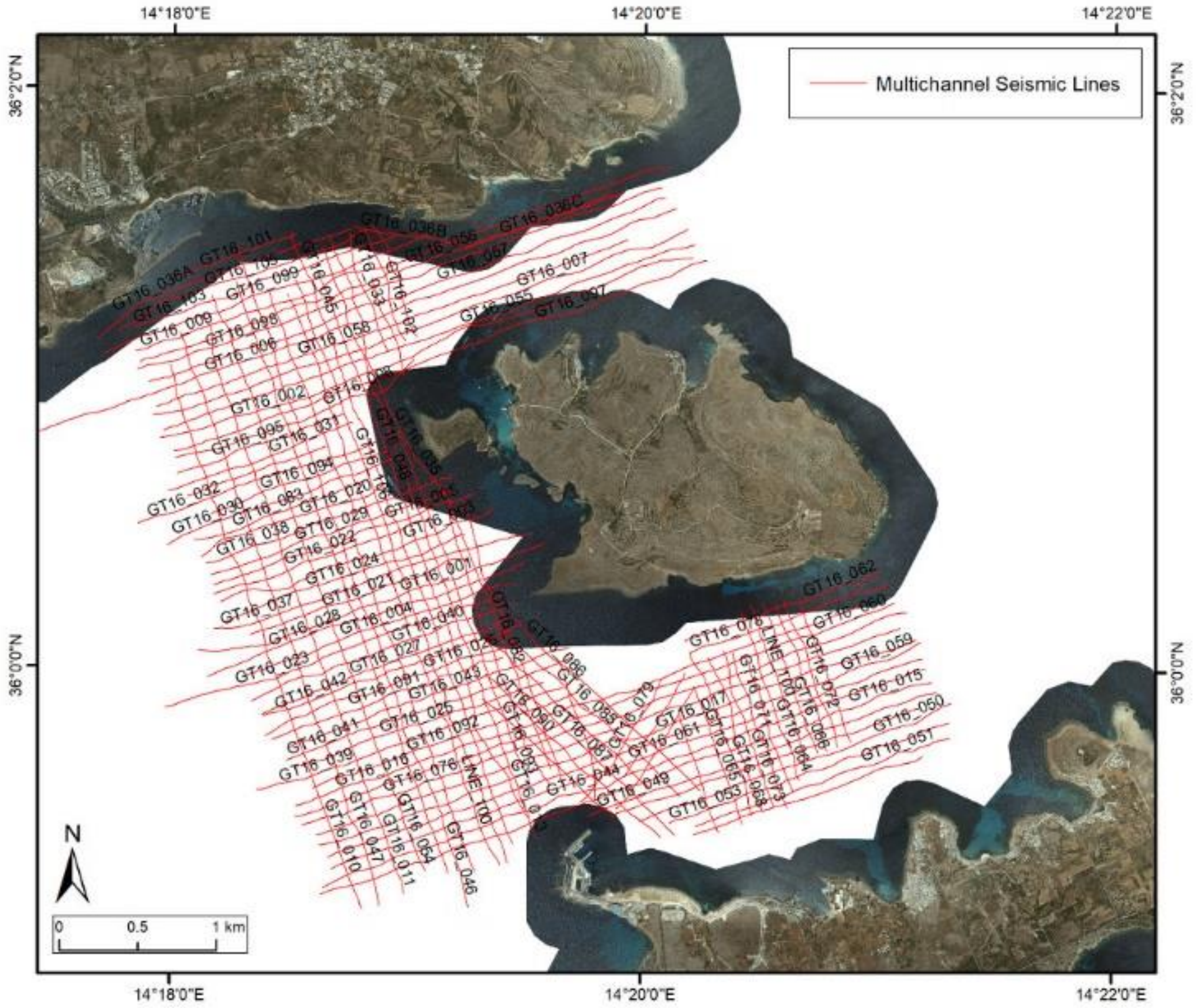
14°18'0"E
14°20'0"E
14°22'0"E
36°2'0"N
36°0'0"N
Multichannel Seismic Lines
N
0
0.5
1 km

Figure S2

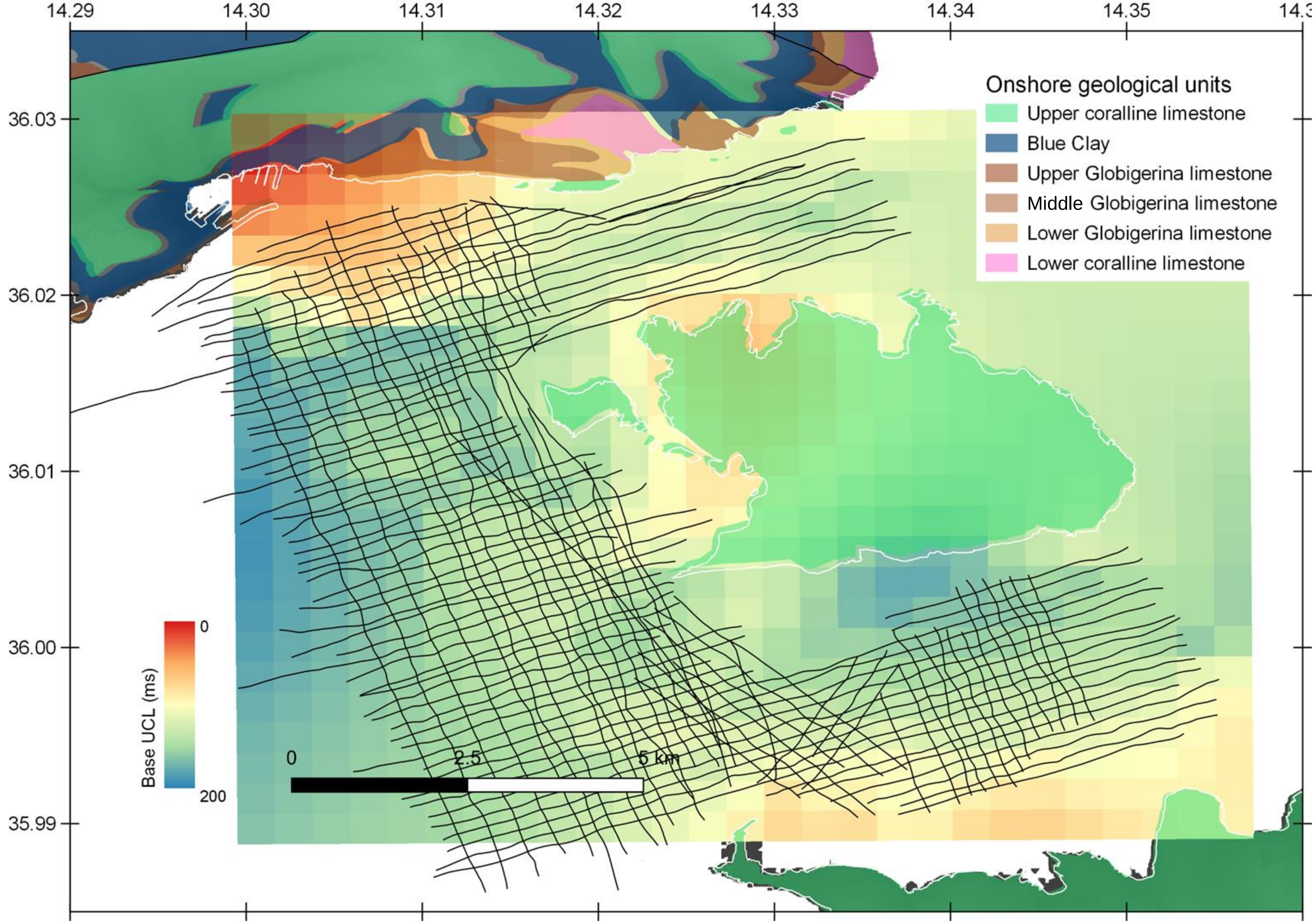
14.29
14.30
14.31
14.32
14.33
14.34
14.35
36.03
36.02
36.01
36.00
35.99
Onshore geological units
Upper coralline limestone
Blue Clay
Upper Globigerina limestone
Middle Globigerina limestone
Lower Globigerina limestone
Lower coralline limestone
Base UCL (ms)
0
200
0
2.5
5 km

Figure S3

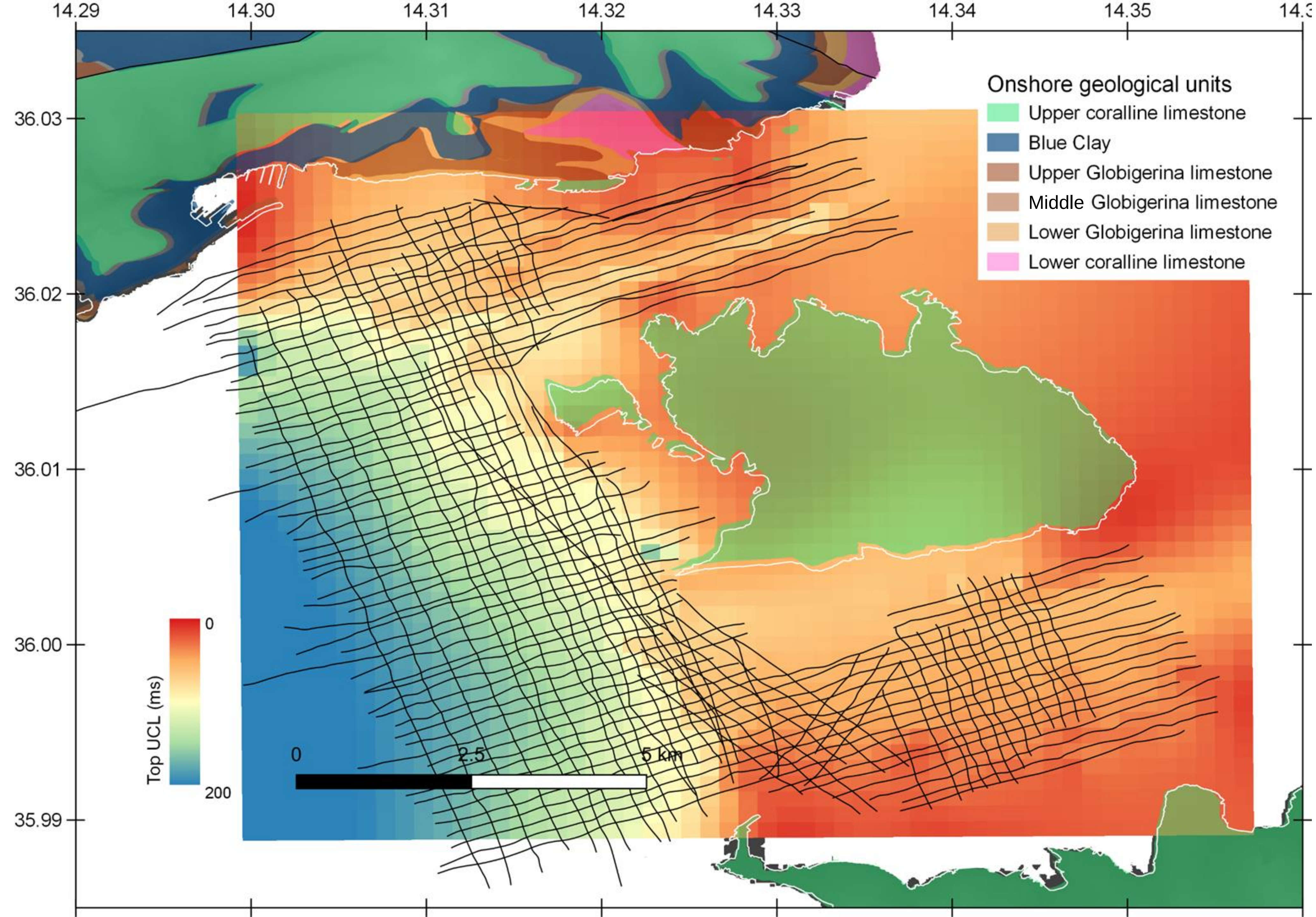
14.29
14.30
14.31
14.32
14.33
14.34
14.35
36.03
36.02
36.01
36.00
35.99
Onshore geological units
Upper coralline limestone
Blue Clay
Upper Globigerina limestone
Middle Globigerina limestone
Lower Globigerina limestone
Lower coralline limestone
Top UCL (ms)
0
200
0
2.5
5 km

Figure S4

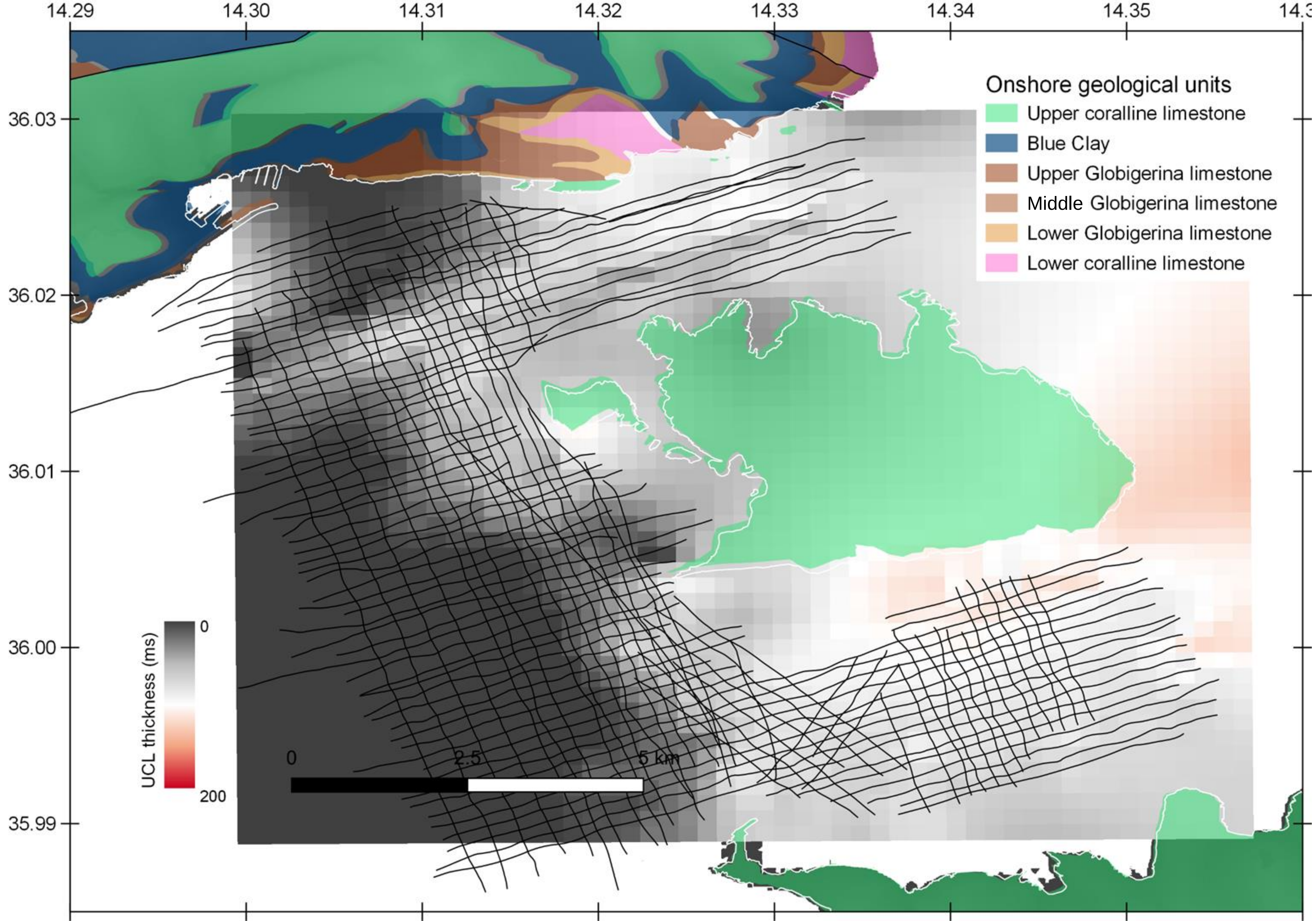

14.29
14.30
14.31
14.32
14.33
14.34
14.35
36.03
36.02
36.01
36.00
35.99
Onshore geological units
Upper coralline limestone
Blue Clay
Upper Globigerina limestone
Middle Globigerina limestone
Lower Globigerina limestone
Lower coralline limestone
UCL thickness (ms)
0
200
0
2.5
5 km
